# Herbacetin Alleviates Influenza Virus-Induced Lung Injury and Fibrosis by Targeting the Neuraminidase Protein

**DOI:** 10.3390/ph18091306

**Published:** 2025-08-30

**Authors:** Feng Liao, Sha Li, Liumei Wu, Jiafan Chen, Ziqing Luo, Ming Zhong, Qiuhong Li, Wenbiao Wang, Geng Li

**Affiliations:** 1 State Key Laboratory of Traditional Chinese Medicine Syndrome, Guangzhou University of Chinese Medicine, Guangzhou 510405, China; feng_liao@yeah.net (F.L.); 20221111054@stu.gzucm.edu.cn (S.L.); wlm521202507@163.com (L.W.); 20221111609@stu.gzucm.edu.cn (J.C.); 20171102364@stu.gzucm.edu.cn (Z.L.); 20221111052@stu.gzucm.edu.cn (M.Z.); 18277115153@163.com (Q.L.); 2 School of Basic Medical Sciences, Southern Medical University, Guangzhou 510515, China

**Keywords:** influenza virus, pulmonary fibrosis, herbacetin, neuraminidase, TGF-β/Smad3 pathway

## Abstract

**Background:** Influenza A virus (IAV) is a major human pathogen, contributing to substantial morbidity and mortality during seasonal outbreaks and pandemics. Human infection with IAV can lead to pneumonia and acute respiratory distress syndrome (ARDS), and numerous clinical and basic research studies have established an association between IAV and pulmonary fibrosis (PF). However, the treatment of IAV-induced PF fibrosis has not been studied and discussed. **Methods:** An IAV-induced PF mouse model was established. Herbacetin (HBT) was identified as the most effective compound in the in vitro study of seven components of *Rhodiola rosea* L. (*R. rosea* L.). The effect of HBT on IAV-induced lung injury and PF was evaluated in vivo and in vitro. The binding between HBT and neuraminidase (NA) protein was investigated by biological layer interferometry (BLI) and cell thermal shift assay (CETSA). **Results:** Following IAV infection, the TGF-β/Smad3 pathway is activated, leading to the upregulation of fibrosis-related proteins that promote fibrosis. HBT exhibited a significant ability to reduce influenza virus-induced lung injury and fibrosis both in vitro and in vivo. Mechanistically, HBT binds to the NA protein of the influenza virus, reducing viral infection and the activation of the TGF-β/Smad3 pathway, thereby mitigating the formation of lung injury and PF. **Conclusions:** HBT represents a promising therapeutic agent for modulating influenza virus-induced lung injury and PF, marking a significant step toward the development of effective treatments for influenza-induced PF.

## 1. Introduction

Influenza A virus (IAV) is a persistent zoonotic pathogen of considerable public health concern. It is estimated that influenza contributes to approximately 300,000 respiratory-related deaths annually worldwide, with the majority occurring in low- and middle-income regions, particularly in sub-Saharan Africa and Southeast Asia, where the burden of influenza-associated mortality is most pronounced [1,2]. Although influenza can affect individuals of all age groups, specific populations, including pregnant women, young children, adults aged 65 years and older, and individuals with pre-existing medical conditions, are at an increased risk of experiencing severe complications [3]. One study indicates that approximately two-thirds (67%) of deaths associated with seasonal influenza occur among individuals aged 65 years and older [4]. In 2018, in children under 5 years, approximately 110 million cases of influenza were reported globally, with an estimated 870,000 resulting in hospitalization [5]. Severe IAV infections often lead to pneumonia and acute respiratory distress syndrome (ARDS) [6]. However, they may also result in pulmonary fibrosis (PF), a progressive condition characterized by excessive extracellular matrix deposition in the lung [7]. Emerging evidence suggests that virus-induced PF develops through inflammatory responses and activation of fibrosis-related pathways, with pathological studies revealing that ARDS patients progress to PF during recovery, highlighting its underrecognized risk following severe IAV infection [8]. Clinical observations since the 2009 H1N1 pandemic have consistently linked IAV to PF, with autopsies revealing diffuse alveolar damage, inflammatory infiltration, fibrosis, and pneumocyte hyperplasia [9]. A longitudinal CT study by Mineo et al. demonstrated that 10% of influenza-associated ARDS patients developed PF, including fatal cases and delayed-onset fibrosis [10]. Similarly, a study in Zhengzhou (2018–2020) identified 232 H1N1-infected ARDS patients with radiologically confirmed PF, while a larger cohort of 14,936 influenza patients revealed a significantly elevated PF risk in ARDS cases [11,12]. Notably, avian influenza strains (H7N9) also induce PF [13], indicating their broad association with influenza virus subtypes. Preclinical models corroborate these findings, showing that H1N1-infected mice develop collagen deposition and alveolar epithelial damage resembling human PF pathology, particularly in aged mice with impaired epithelial regeneration [14,15]. These findings support the use of IAV-infected mice as a relevant model to study the pathogenesis of viral PF.

The pathogenic mechanisms driving influenza virus-induced PF remain incompletely elucidated, particularly regarding the dual roles of NA in viral pathogenesis and fibrotic progression [16,17]. As a critical viral surface protein, NA mediates receptor destruction through sialic acid cleavage, facilitating viral particle release and sustaining infection cycles [18]. Additionally, NA has been shown to interact with latent transforming growth factor-β (TGF-β), promoting its maturation and activating downstream signaling pathways [14,19]. The TGF-β/Smad3 pathway is the principal signaling involved in the regulation of PF [20]. This process involves the binding of TGF-β to its receptor, phosphorylation of the downstream Smad3 protein, and subsequent nuclear translocation of Smad3 to regulate the transcription and expression of fibrosis-related genes, such as fibronectin (Fn), Snail, and collagen I, thereby contributing to the onset and progression of PF [21,22]. Concurrently, IAV infection triggers a robust inflammatory milieu that synergistically exacerbates fibrotic processes. The early recruitment of innate immune cells, particularly macrophages and neutrophils, to infected lung tissue generates a cytokine storm characterized by elevated IL-1β, IL-6, and tumor necrosis factor-α (TNF-α) [23]. These pro-inflammatory mediators amplify tissue damage and create a permissive environment for TGF-β activation, thereby bridging acute inflammation with chronic fibrotic remodeling [24]. The pathogenic interplay between viral components, particularly NA, and the host inflammatory response highlights the complex molecular pathogenesis of IAV-related PF.

Current therapeutic options for influenza-induced PF remain limited, with clinical management primarily relying on antiviral agents like neuraminidase inhibitor oseltamivir (OSE) and RNA polymerase inhibitor favipiravir to control viral replication [25,26]. Although current influenza vaccines are effective against well-matched viral strains, new vaccines must be developed each season due to antigenic drift and shift. Additionally, a sufficient quantity of vaccines targeting emerging subtypes often requires time to become available [27]. While paeoniflorin has demonstrated efficacy in mitigating influenza-associated PF in preclinical models, the persistent threat of seasonal influenza outbreaks underscores the urgent need for more effective treatments against virus-induced fibrotic complications. *Rhodiola rosea* L. (*R. rosea* L.), commonly known as “golden root” or “roseroot”, is a member of the plant family Crassulaceae and is known for its diverse protective effects, including anti-diabetic, anti-cancer, anti-aging, anti-inflammatory, and immune-regulatory properties [28,29]. *R. rosea* L. has emerged as a valuable medicinal plant in both traditional and contemporary medicine across numerous countries, including Sweden, Norway, France, Germany, Russia, and China [30]. As a traditional Chinese medicinal herb, *R. rosea* L. has been clinically utilized to treat various lung diseases, including lung injury and PF [31]. Studies have demonstrated its efficacy in treating bleomycin-induced PF in mice [32]. Additionally, *R. rosea* L. has exhibited inhibitory effects against the H3N2 influenza virus, suggesting that its active compounds may have the potential to treat influenza-induced PF [33]. Currently, researchers have isolated over 140 compounds from *R. rosea* L., predominantly flavonoids, coumarin, volatiles, anthraquinones, and organic acids [34], including rhodioloside, HBT, and rosavin [35]. Herbacetin (HBT), a flavonoid, serves as a quality control marker for *R. rosea* L. [36]. HBT has demonstrated a variety of biological activities, including anti-inflammatory effects through inhibition of the NF-κB pathway, alleviation of myocardial hypertrophy by targeting the SGK1 protein [37], and potential activity against SARS-CoV-2, as predicted by bioinformatics analysis [38]. However, to date, no studies have investigated its potential for treating lung injury and PF induced by influenza virus infection.

In our study, we investigated the progression of PF following influenza virus infection in mice by analyzing fibrotic phenotypes and the expression of fibrosis-related proteins in lung tissues. We identified HBT, a compound derived from *R. rosea* L., as a significant attenuator of influenza-induced lung injury and PF, both in vitro and in vivo. Mechanistically, HBT binds to the NA protein of the influenza virus, effectively inhibiting NA enzyme activity and reducing viral infection, thereby alleviating pathological changes in lung tissue. Furthermore, HBT inhibited the TGF-β/Smad3 signaling pathway and attenuated the progression of PF. This study underscores the association between influenza infection and PF progression, enhances our understanding of virus-induced PF, and identifies NA proteins as potential therapeutic targets. Our findings also suggest that HBT is a promising pharmacological intervention for mitigating influenza virus-associated PF.

## 2. Results

### 2.1. IAV Infection Induces Expression of Fibrotic Proteins to Promote PF In Vivo

**Figure 1:**
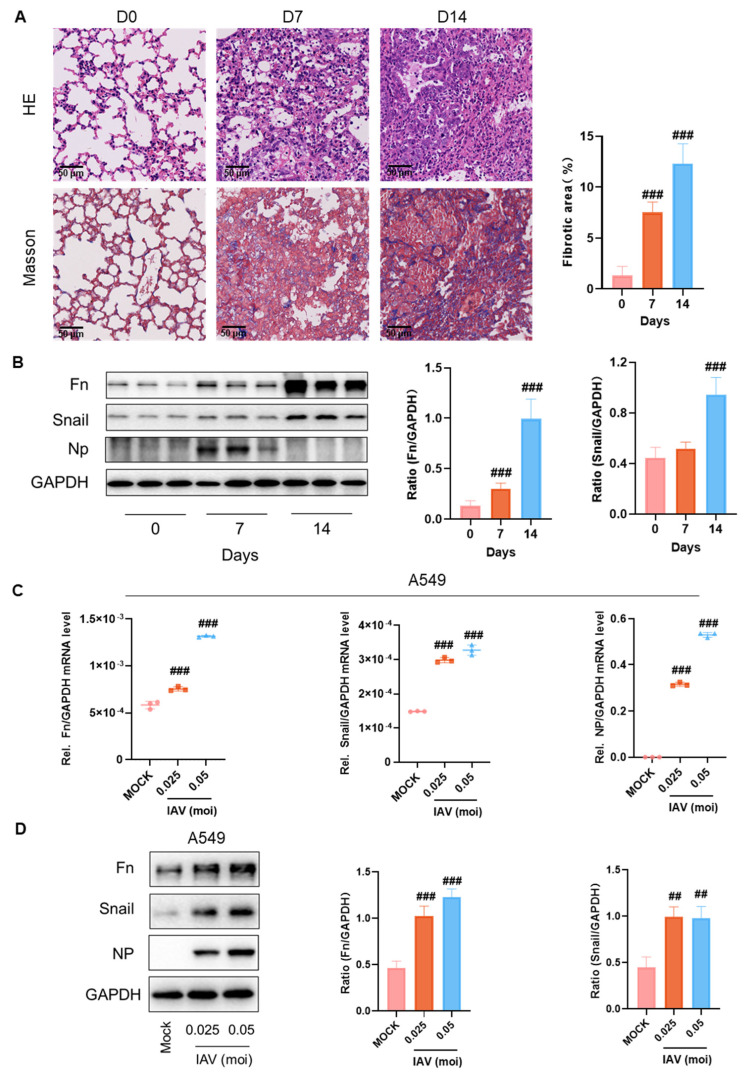
IAV infection promotes the expression of fibrotic proteins and induces PF. (**A**) HE (top) and Masson (bottom) staining were performed on mouse lung tissue, with statistical analysis of fibrotic areas shown on the right. (**B**) Western blot analysis was used to measure the protein expression levels of Fn, Snail, and NP in lung tissue of mice. (**C**) The mRNA levels of Fn and Snail in A549 cells were measured using qPCR 24 h after infection with IAV at different MOIs. (**D**). After A549 cells were infected with different MOIs of IAV for 30 h, the expression of Fn and Snail proteins in A549 cells infected with IAV was detected by Western blot. ##, *p* < 0.01; ###, *p* < 0.005; Versus mock.

Influenza virus-induced PF has been reported in clinical settings, and further animal studies are warranted. The commonly used H1N1 (PR8) strain was used to infect BALB/c mice, based on our previous influenza animal model and references from other studies [14,39]. The development of PF following IAV infection was investigated by collecting lung tissue from mice at 7 and 14 days post-infection. HE staining revealed severe lung tissue damage on both day 7 and day 14 post-infection (Figure 1A, top panel). Masson’s trichrome staining, a key method for assessing PF, highlighted blue collagen deposition in the lung tissue. Collagen accumulation was observed on both days 7 and 14, with fibrosis more pronounced on day 14 (Figure 1A, bottom panel). Levels of fibrotic-related proteins Fn and Snail were measured, and increased protein levels were detected in lung tissue from mice infected for 14 days (Figure 1B).

Lung epithelial cells are an important cell population in the development of lung injury and fibrosis. We further examined the mRNA levels of PF-related genes in A549 cells after IAV infection. The results depicted in Figure 1C reveal elevated mRNA levels of Fn and Snail, indicating that transcription of fibrosis-associated genes has commenced. Finally, the protein expression levels of Fn and Snail in the cells were examined. Consistent with the results in animal tissues, infection of A549 cells with the virus resulted in increased expression of fibrosis-related proteins (Figure 1D). These findings revealed that lung tissue from mice infected with the influenza virus progressed from injury to PF by 14 days post-infection.

### 2.2. IAV Infection Activates the TGF-β/Smad3 Pathway Both In Vivo and In Vitro

**Figure 2:**
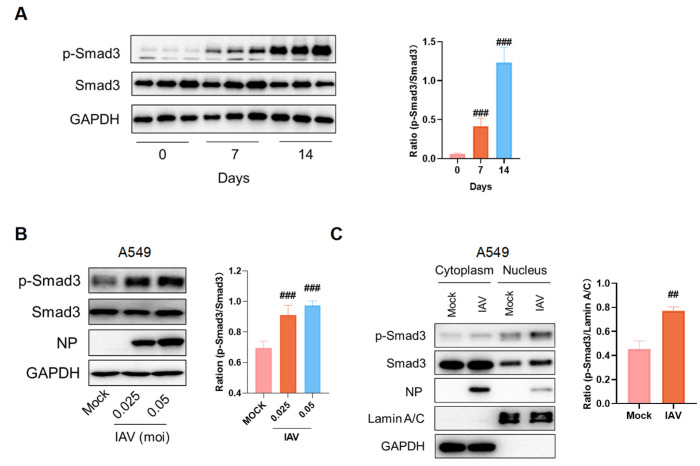
IAV infection activates the TGF-β/Smad3 pathway. (**A**) Western blot analysis was performed to assess the phosphorylation levels of Smad3 protein in lung tissue from mice in each group. (**B**) The phosphorylation of Smad3 protein in A549 cells was measured 16 h after infection with IAV at various MOIs. (**C**) A549 cells were infected with IAV (MOI = 0.05) for 18 h to perform a nuclear and cytoplasmic extraction assay. ##, *p* < 0.01; ###, *p* < 0.005; Versus mock.

The TGF-β/Smad3 pathway is a critical signaling cascade involved in the regulation of PF. Changes in proteins associated with this pathway were assessed. Our results revealed a significant increase in Smad3 phosphorylation levels on day 14 post-infection (Figure 2A). RNA sequencing results of lung tissues from influenza-infected mice from the GEO database were analyzed, and it was found that genes related to fibrosis and the TGF-β pathway were upregulated (Figure S1). Subsequently, alterations in Smad3 levels in cells following influenza virus infection at varying MOIs were examined. Figure 2B shows that Smad3 phosphorylation was elevated in A549 cells post-infection, indicating activation of the TGF-β/Smad3 pathway. Phosphorylated Smad3 functions as a transcription factor and translocates to the nucleus. Therefore, Smad3 levels in the nucleus were assessed. As depicted in Figure 2C, phosphorylated Smad3 levels increased within the nuclei following viral infection. These findings indicate that influenza virus infection can activate the TGF-β/Smad3 pathway and promote PF.

### 2.3. HBT Anti-Fibrosis Induced by IAV In Vitro

**Figure 3:**
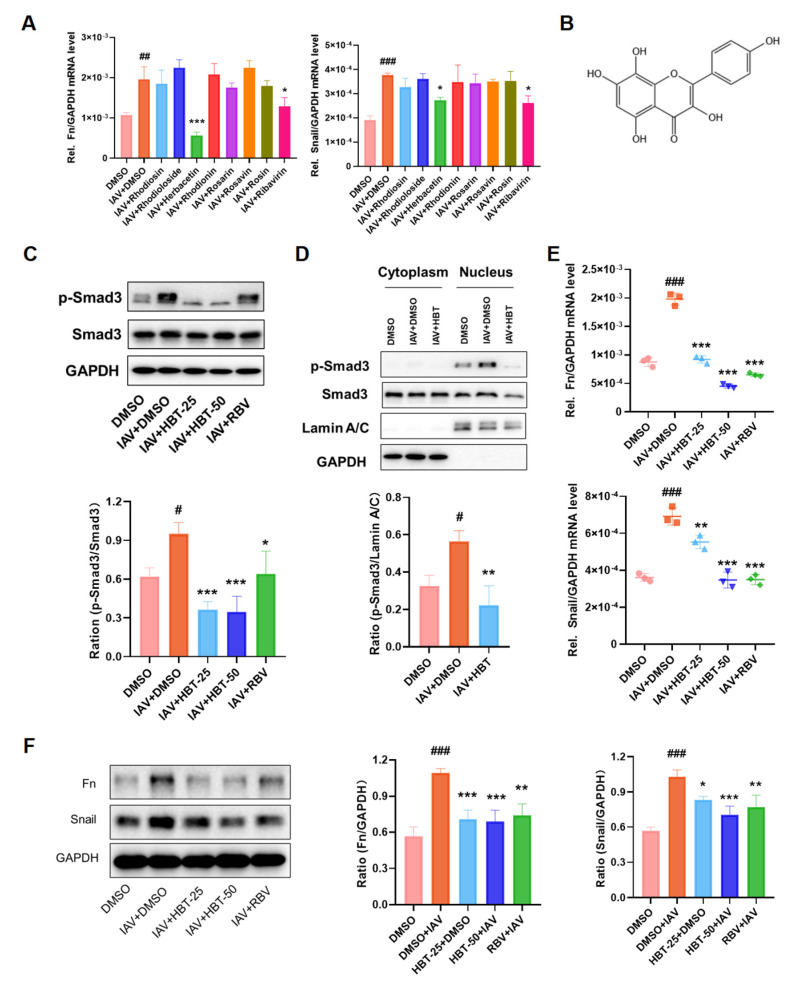
HBT inhibited IAV-induced activation of the TGF-β/Smad3 pathway and fibrosis markers in vitro. (**A**) Seven active compounds from *R. rosea* L. were screened for their anti-influenza fibrosis efficacy in A549 cells, with a drug concentration of 50 μM and RBV as the positive control. After infection with IAV (MOI = 0.05), mRNA levels of Fn and Snail were measured 24 h post-treatment. (**B**) The chemical structure of HBT was presented. (**C**) The phosphorylation level of Smad3 was assessed in A549 cells treated with HBT at concentrations of 25 and 50 μM for 16 h after viral infection. (**D**) Phosphorylation levels of Smad3 in the nucleus were evaluated in A549 cells 18 h post-viral infection. (**E**) The effect of HBT on mRNA levels of Fn and Snail was analyzed in A549 cells 24 h after viral infection. (**F**) The impact of HBT on protein levels of Fn and Snail was determined in A549 cells 30 h post-infection. #, *p* < 0.05; ##, *p* < 0.01; ###, *p* < 0.005; Versus DMSO and *, *p* < 0.05; **, *p* < 0.01; ***, *p* < 0.005; Versus DMSO+IAV.

*R. rosea* L. has demonstrated significant pharmacological activity in the treatment of PF. To investigate which major active compounds of *R. rosea* L. exert an inhibitory effect on influenza virus-induced PF, we initially evaluated seven principal active compounds in vitro. The results presented in Figure 3A indicate that HBT exhibited the best antifibrotic efficacy among these active compounds. The chemical structure of HBT, along with the structures of the other compounds, is illustrated in Figure 3B. Based on our initial screening results, we selected HBT as the focus for further experimental studies.

The toxicity of HBT on A549 cells was assessed, with the results shown in Figure S2 indicating that the CC_50_ of HBT for A549 cells exceeded 200 μM. Previously, we confirmed that influenza virus infection activates the TGF-β/Smad3 signaling pathway. Therefore, the effect of HBT on Smad3 protein levels was evaluated. Our findings demonstrated that HBT significantly decreased the phosphorylation of Smad3 (Figure 3C) and inhibited its nuclear translocation (Figure 3D). Nuclear translocation of Smad3 can activate the transcription and expression of fibrosis-related genes. Consequently, we measured the mRNA and protein levels of Fn and Snail following HBT treatment. The results showed that a concentration of 50 µM HBT notably reduced both the mRNA (Figure 3E) and protein levels (Figure 3F) of Fn and Snail. These findings indicate that HBT inhibits the IAV-activated TGF-β/Samd3 pathway in vitro and inhibits downstream fibrotic proteins. These findings also revealed that HBT inhibited TGF-β/Samd3 signaling and thus inhibited the progression of fibrosis in vitro.

### 2.4. HBT Inhibited TGF-β-Induced Fibrosis Markers In Vitro

**Figure 4:**
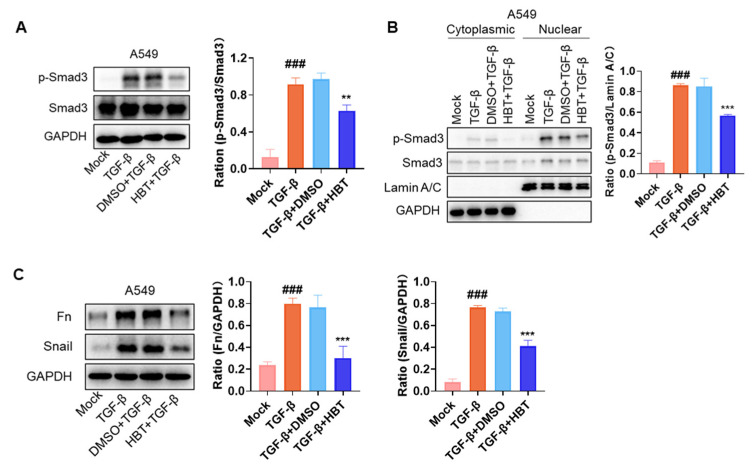
HBT inhibits the TGF-β/Smad3 pathway and expression of fibrotic proteins induced by TGF-β. (**A**) Western blot analysis was used to detect the phosphorylation of Smad3 in A549 cells. HBT was added to the cells 6 h prior to stimulation with TGF-β (5 ng/mL) for 1 h. (**B**) Western blot analysis was also employed to assess the nuclear translocation of Smad3 in A549 cells. After incubating with HBT for 6 h, cells were stimulated with TGF-β (5 ng/mL) for 2 h, followed by collection for nuclear and cytoplasmic extraction experiments. (**C**) Western blot was used to evaluate the expression levels of Fn and Snail proteins in A549 cells. Six hours after adding HBT, TGF-β (10 ng/mL) was added, and cells were stimulated for 24 h. ###, *p* < 0.005; Versus Mock and **, *p* < 0.01; ***, *p* < 0.005; Versus TGF-β+DMSO.

The activation of the TGF-β/Smad3 pathway by IAV infection is a critical factor in the development of PF, with TGF-β playing a key role in this process. In vitro stimulation of cells with TGF-β is a common approach for studying antifibrotic drugs. The activation of the TGF-β/Smad3 pathway comprises several critical steps. First, TGF-β induces the phosphorylation of Smad3. Subsequently, Smad3 translocates to the nucleus, where it regulates mRNA transcription. Finally, Smad3 modulates the expression of fibrosis-related proteins. Based on this, TGF-β protein stimulation at different time points was used for sample collection and analysis. The inhibitory effects of HBT on TGF-β-induced signaling in A549 cells were then assessed. The results demonstrated that HBT inhibited TGF-β-induced Smad3 phosphorylation (Figure 4A) and nuclear translocation (Figure 4B). Additionally, HBT suppressed the expression of fibrosis-related proteins induced by TGF-β (Figure 4C). These findings indicate that HBT not only inhibits the TGF-β/Smad3 pathway activated by IAV but also directly interferes with the TGF-β signaling pathway.

### 2.5. HBT Reduced Lung Injury and PF Induced by IAV In Vivo

**Figure 5:**
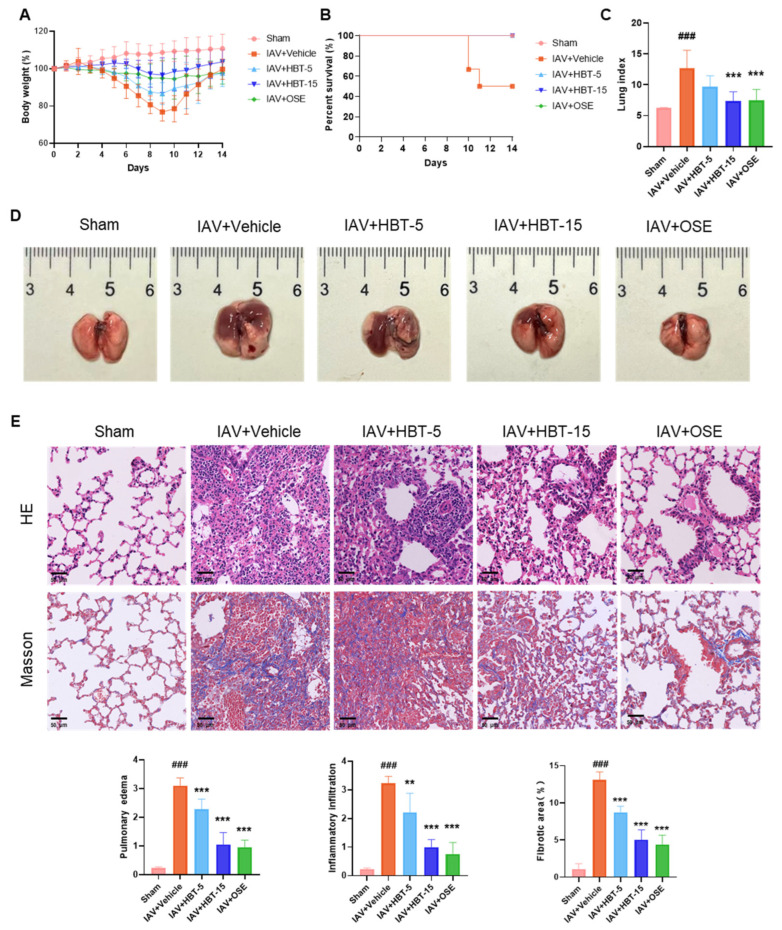
HBT alleviates lung injury and PF induced by the influenza virus in vivo. (**A**) Results of changes in body weight of mice in each group, calculated with Day 0 as the initial body weight. (**B**) Mortality of mice in each group. (**C**) Lung index of mice in each group. (**D**) Tissue map of the mouse lung. (**E**) HE staining and Masson staining results of lung tissue of mice in each group; the inflammatory infiltration and edema of mice in each group were scored, and the PF area was calculated. ###, *p* < 0.005; Versus Sham and **, *p* < 0.01; ***, *p* < 0.005; Versus IAV+Vehicle.

To further elucidate the inhibitory effect of HBT on influenza virus-induced PF, a 14-day animal model of IAV infection was employed for drug evaluation. In terms of body weight change, the effect of high doses of HBT was comparable to OSE and did not cause much weight loss after viral infection (Figure 5A). Neither HBT nor OSE caused mortality in the mice (Figure 5B). Lung index data showed that high-dose HBT treatment significantly reduced the increase in the lung index (Figure 5C). Images were taken to document the extent of damage observed in the mice (Figure 5D). Subsequently, HE staining was performed on lung tissues to assess inflammatory infiltration, pulmonary edema, and tissue injury. Statistical analysis of the staining across groups is presented in Figure 5E, showing that high-dose HBT exerted a pharmacological effect on inflammatory infiltration and pulmonary edema nearly equivalent to OSE, whereas low-dose HBT demonstrated slightly reduced efficacy. Masson staining was used to evaluate fibrotic changes, revealing collagen deposition in the lungs following influenza virus infection, consistent with previous findings. However, both high-dose HBT and oseltamivir treatment markedly reduced collagen deposition (Figure 5E, down). Statistical data on inflammatory infiltration, pulmonary edema, and fibrotic area are provided below. These results indicate that HBT reduces lung injury and PF induced by IAV in vivo.

### 2.6. HBT Reduced TGF-β/Smad3 Activation and Expression of Fibrosis-Related Proteins In Vivo

**Figure 6:**
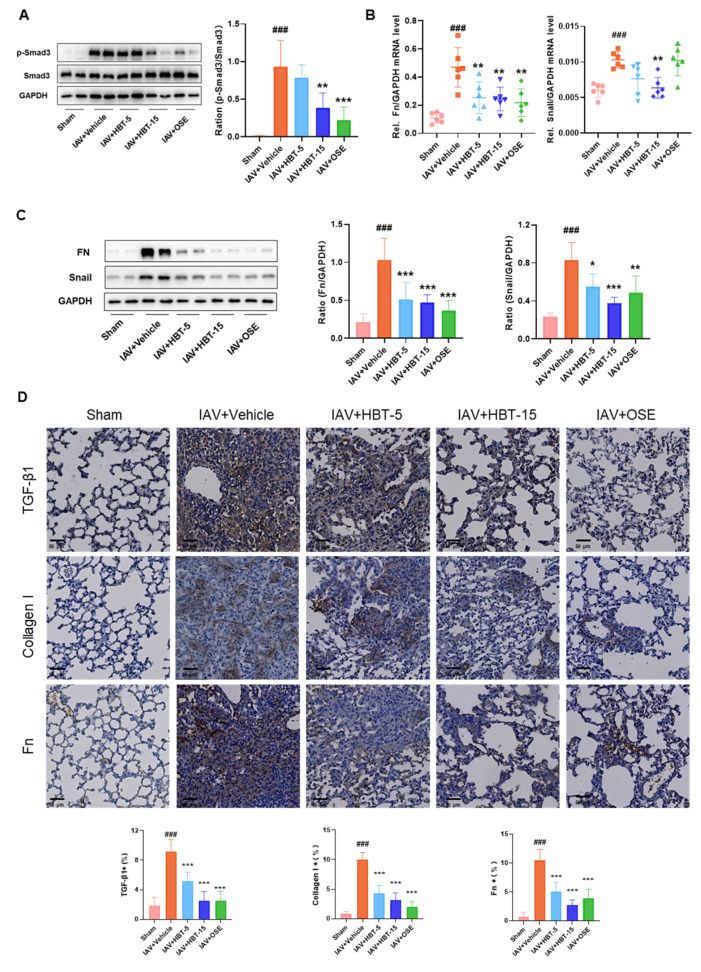
HBT alleviates PF by modulating TGF-β/smad3 activation. (**A**) The protein levels of phosphorylated Smad3 and total Smad3 in lung tissues from various groups at 14 days post-influenza virus infection are presented, with the right panel displaying the corresponding statistical results. (**B**) mRNA expression levels of Fn and Snail in lung tissues from different mouse groups. (**C**) The protein levels of Fn and Snail in the lung tissues across all groups of mice were detected. (**D**) Results from immunohistochemical analysis for TGF-β1, collagen I, and Fn in the lung tissues from each group of mice are provided, accompanied by relevant statistical data below. ###, *p* < 0.005; Versus Sham and *, *p* < 0.05; **, *p* < 0.01; ***, *p* < 0.005; Versus IAV+Vehicle.

Previously, we discovered that fibrosis induced by influenza virus infection was associated with the TGF-β/Smad3 signaling pathway. Consequently, we investigated the phosphorylation status of Smad3 protein in the lung tissue of mice and found that HBT administration significantly reduced the phosphorylation level of Smad3 (Figure 6A). Subsequently, we assessed both mRNA levels (Figure 6B) and protein quantities (Figure 6C) of Fn and Snail. The results indicated that both high-dose and low-dose groups of HBT, as well as the OSE group, effectively mitigated the increase in Fn protein expression and mRNA levels. Notably, for Snail, OSE exhibited a lesser inhibitory effect compared to the high-dose HBT group. The immunohistochemical staining of lung tissue revealed a significant increase in type I collagen in the virus-infected group, which is consistent with our observations from the Masson staining. Additionally, TGF-β1, collagen I, and Fn proteins were markedly increased in the virus group. However, both high-dose and low-dose groups of HBT, along with the OSE group, demonstrated reductions in these proteins within lung tissue (Figure 6D). Results showed that HBT also inhibited the TGF-β/Smad3 pathway and reduced the expression of downstream fibrosis proteins in vivo.

### 2.7. HBT Reduces IAV Infection by Blocking the NA Protein

**Figure 7:**
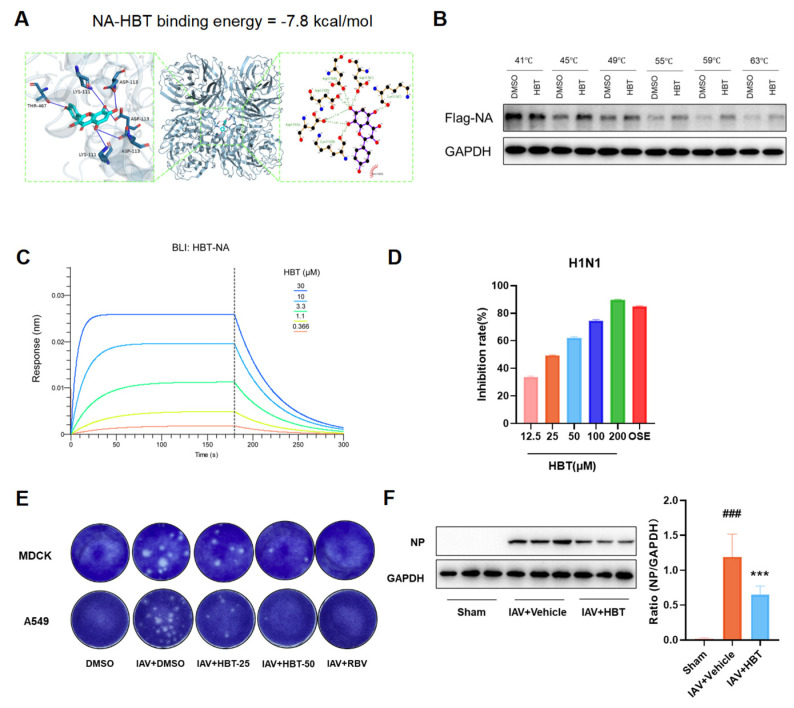
HBT reduces IAV replication by binding to NA proteins. (**A**) Molecular docking analysis of HBT and the N1 protein. (**B**) CETSA results for NA proteins. The Flag-NA plasmid (8 μg) was transfected into HEK293T cells for CETSA analysis, measuring the levels of Flag-NA and GAPDH in both DMSO and HBT (100 μM) treatment groups at various temperatures. (**C**) BLI detection of HBT binding to the N1 protein in vitro. (**D**) Effect of HBT on NA (H1N1) protease activity. The H1N1 (PR8) virus was inactivated and used for enzyme inhibition assays. HBT was diluted with DMSO to prepare different concentrations for detection. OSE at a concentration of 10 μM was used as a positive control. (**E**) Viral titers in supernatants from MDCK and A549 cells were determined by plaque assay. HBT was added to MDCK and A549 cells 2 h prior to IAV (MOI = 0.1) infection. After 2 h of infection, the cells were cultured for an additional 48 h, and the supernatant was collected for plaque detection. (**F**) Western blot analysis was used to detect NP protein expression in the lung tissues of mice infected for 7 days. ###, *p* < 0.005; Versus Sham and ***, *p* < 0.005; model Versus IAV+Vehicle.

NA of the influenza virus can promote the activity of TGF-β and activate the TGF-β/smad3 signaling pathway to promote the formation of PF. At the same time, the NA protein plays an important role in the invasion of the influenza virus and the release of virus particles. We simulated the molecular docking between the NA protein and HBT, and the results showed that HBT might bind to the NA protein, and the binding energy of −7.8 kcal/mol indicates a strong affinity between HBT and N1, with binding energies below −5.0 kcal/mol (Figure 7A). We conducted CETSA experiments, and the NA protein underwent degradation as the temperature increased. However, the rate of decrease in NA protein levels within the HBT treatment group was slower compared to that observed in the DMSO group (Figure 7B). To further determine the interaction between the NA protein and HBT, we used a BLI assay for validation. The results presented in Figure 7C indicate that HBT and NA proteins interact directly in vitro, with an equilibrium association constant (KD) of 5.702 μM. This reveals a strong binding affinity between HBT and the NA protein. The association rate constant (Kon) for the interaction between HBT and NA proteins is 4.241 × 10^3^ Ms^−1^, while the dissociation rate constant (Koff) is 2.418 × 10^-2^ S^−1^. These results indicate that HBT is able to bind to NA proteins.

The effect of HBT on viral infection after binding to the NA protein was further investigated. The impact of HBT on NA protease activity was assessed, and the results shown in Figure 7D indicate that HBT inhibits N1 enzymatic activity in a concentration-dependent manner. Additionally, enzyme activity inhibition tests conducted on NA proteins from various viruses revealed that HBT exerts an inhibitory effect (Figure S3). In vitro, HBT intervention in IAV-infected MDCK and A549 cells resulted in a reduction of progeny viral particles in the supernatant (Figure 7E). To further validate these findings, the expression of influenza virus NP protein in lung tissue was examined, and it was found that HBT reduced IAV replication in the lung (Figure 7F). These results suggest that HBT diminishes influenza virus replication and activation of the TGF-β/Smad3 pathway by targeting the NA protein and inhibiting its enzymatic activity, which ultimately mitigates lung injury and the progression of PF.

## 3. Discussion

In this study, we demonstrate that IAV infection induces PF both in vivo and in vitro, which is closely associated with the TGF-β/Smad3 signaling pathway. HBT exhibits significant antifibrotic effects, as confirmed in both in vivo and in vitro studies. HBT directly binds to the influenza virus NA protein, and this interaction not only alleviates PF mediated by the TGF-β/Smad3 pathway but also inhibits viral replication. These findings underscore the therapeutic potential of HBT for treating PF associated with influenza and, further, emphasize the NA protein as a critical target for managing diseases caused by influenza virus infection.

Lung injury, pneumonia, and acute respiratory distress syndrome (ARDS) caused by influenza virus infection are well-recognized complications. However, the long-term effects of influenza virus infection are often underestimated. Influenza virus-induced PF represents a form of chronic damage resulting from the disease, which has been clinically observed [7,8]. Nonetheless, animal models of influenza virus-induced PF require further investigation. A previous study reported collagen deposition in the lung tissue of C57BL/6NJ mice infected with the H1N1 (PR8) virus on day 15 [12]. In our study, BALB/c mice were infected with the H1N1 (PR8) virus. Severe tissue damage and collagen deposition were observed in the lung tissues of BALB/c mice on day 14. Additionally, activation of the TGF-β/Smad3 pathway and increased expression of downstream PF-related proteins were noted. These findings suggest that our mouse model exhibits a phenotype of PF, which can be utilized for drug evaluation.

Currently, the primary drugs used to treat influenza virus infections include oseltamivir, zanamivir, baloxavir, and so on. These inhibitors have been shown to alleviate symptoms of lung injury and pneumonia caused by influenza. However, the effects of these drugs on influenza virus-induced PF remain unclear. Furthermore, the efficacy of medications used to treat PF, such as pirfenidone and nintedanib, in addressing influenza-induced PF is also unknown. Therefore, identifying drugs capable of modulating influenza-induced PF is of significant importance. HBT has been reported to have beneficial effects on multiple tissues and organs. Previous studies have shown that HBT can inhibit angiogenesis in malignant melanoma by blocking the EGFR-ERK/AKT signaling pathway [40]. Additionally, HBT has been shown to inhibit asthma development by blocking the SGK-1/NF-κB signaling pathway [41]. However, the impact of HBT on PF has not yet been explored. In our study, we observed that HBT improved PF in vitro and in vivo by reducing influenza virus infection. Moreover, HBT was found to reduce fibrosis markers induced by TGF-β in vitro. These findings suggest that HBT exhibits dual “antiviral and antifibrotic” effects, which holds significant therapeutic potential. Influenza virus infection first occurs through viral invasion and replication, followed by lung damage [42]. After the acute phase, the body enters a repair phase, but abnormal repair processes can lead to fibrosis [12]. During these processes, HBT can reduce acute lung injury caused by influenza virus infection. Additionally, HBT has the potential to improve long-term chronic lung diseases, offering more than just a single therapeutic effect.

HBT is a flavonoid, and its structure closely resembles that of other reported antiviral and antifibrotic flavonoids, such as quercetin and kaempferol. This structural similarity contributes to its therapeutic effects. However, like most flavonoids, HBT faces challenges, such as low bioavailability and poor solubility [43]. Ge et al. investigated the pharmacokinetics of HBT in rats and found that its clearance rate and half-life were 16.4 ± 1.92 mL/kg⋅min and 1.32% [44]. These pharmacokinetic properties limit its practical use as a drug. Nonetheless, strategies such as crystal engineering, solid dispersion technology, and nanotechnology can be employed to improve the formulation, enhancing its solubility and bioavailability and thereby increasing its clinical applicability [45]. There are several limitations to the current study. First, the potential toxicities and side effects of HBT were not evaluated, and the safety concerns associated with its use were not addressed. Second, the antifibrotic effects of HBT were not compared with those of established antifibrotic agents, such as pirfenidone. Third, clinical samples from patients infected with the influenza virus were not examined, and the involvement of the TGF-β/Smad3 signaling pathway in influenza-induced PF remains unexplored. Future studies addressing these aspects are essential to broaden the clinical application of HBT and to further elucidate its mechanism in treating virus-induced PF.

## 4. Materials and Methods

### 4.1. Cells and Virus

Human embryonic kidney (HEK293T) cells and Human lung adenocarcinoma 549 (A549) cells were purchased from the American Type Culture Collection. MDCK cells were donated from the State Key Laboratory of Respiratory Disease. HEK293T cells and MDCK cells were cultured in Dulbecco’s modified Eagle’s medium (DMEM) (Gibco, Grand Island, NY, USA), supplemented with 10% FBS, 100 U/mL penicillin, and 100 μg/mL streptomycin sulfate. A549 cells were cultured using Dulbecco’s modified Eagle’s medium/Nutrient Mixture F-12 (DMEM/F12) (Gibco, Grand Island, NY, USA) containing 10% FBS and 1% penicillin/streptomycin (P/S). The cells were cultured in an incubator at 37 °C and 5% CO_2_.

Influenza A/Puerto Rico/8/34 (PR8, H1N1), A/Chicken/Guangdong/1996 (H9N2), A/HongKong/498/97 (H3N2), and influenza B/Lee/1940 viruses were maintained at the Laboratory Animal Center, Guangzhou University of Chinese Medicine. The viruses were amplified using eggs and stored in aliquots at −80 °C. Viral titers were measured using a plaque assay.

### 4.2. Compounds

Herbacetin (purity ≥ 98%), rhodioloside (purity ≥ 98%), rhodiosin (purity ≥ 98%), rhodionin (purity ≥ 98%), rosarin (purity ≥ 98%), rosavin (purity ≥ 98%), rosin (purity ≥ 98%), and ribavirin (purity ≥ 98%) were procured from Baoji Herbest Bio (Shannxi, China). OSE was obtained from Roche Pharma (Basel, Switzerland)and MCE (Monmouth Junction, NJ, USA). PMA was obtained from Selleck (Houston, TX, USA). For the experiments, herbacetin and ribavirin were dissolved in a DMSO solution, while oseltamivir phosphate was prepared in a saline solution.

### 4.3. Animal Experiment

Male BALB/c mice, 6–8 weeks old and 18–22 g, were purchased from Zhuhai Bestest Bio-Tech Co., Ltd (Zhuhai, China). Before starting the experiment, the mice were administered adequate water and feed to ensure acclimatization. All animal experiments were approved by the Experimental Animal Ethics Committee of Guangzhou University of Chinese Medicine (Guangzhou, China) (Approval No.: 20240807011).

To investigate the association of IAV with PF, mice were randomly divided into three groups of five mice each. They were, respectively, the day 0 group, day 7 group, and day 14 group. General anesthesia was induced with isoflurane inhalation, and animals were removed from the chamber upon achieving a complete anesthetic state. For the day 0 group, control mice received 50 μL sterile phosphate-buffered saline (PBS) via intranasal administration. The remaining mice were inoculated intranasally with 50 μL viral suspension containing IAV (PR8) (80 pfu/mouse), delivered gradually to ensure proper pulmonary delivery. Lung tissues were collected on days 0, 7, and 14 for subsequent analysis.

To evaluate the efficacy of HBT on PF induced by IAV in mice, we randomly divided the mice into five groups with six mice in each group. They were, respectively, the sham group, the IAV with vehicle group, the IAV with OSE group, and the high-dose and low-dose HBT groups. HBT was dissolved using a 10% (*w/v*) β-cyclodextrin solution to form an inclusion complex to enhance the drug’s stability and solubility. The mice in the drug group were administered HBT by intraperitoneal injection (i.p.) one day before the experiment, and the mice in the low-dose group and the high-dose group were administered HBT at 5 mg/kg/day (HBT-5) and 15 mg/kg/day (HBT-15), respectively. Except for the mice in the sham group, all the other mice were anesthetized with isoflurane and administered IAV (PR8, 80 pfu/mouse) via nasal drops. Each subsequent day, mice were administered a drug or solvent. Mice in the oseltamivir group were administered 19.5 mg/kg of the drug, and the viral group and sham group were administered a solvent. The experiment was terminated on day 7 and day 14, respectively. The blood of the mice was collected for serum separation, the lung weight of the mice was measured, and the lung tissue of the mice was collected for subsequent detection.

### 4.4. Viral Infection Cells and Drug Therapy

HBT or RBV was administered to A549 and MDCK cells two hours prior to infection, after which the medium was replaced with a serum-free medium. The multiplicity of infection (MOI) for IAV infection was set at 0.05 for fibrosis detection and at 0.1 for antiviral detection. Two hours post-infection, the medium was removed, and cells were cultured in medium containing 1% FBS and the respective drug until samples were collected at the designated time points.

### 4.5. Western Blot

The cells were lysed for 1 h using RIPA lysate buffer (P0013B, Beyotime, Shanghai, China). Subsequently, we quantified the protein concentration using a BCA Protein Assay Kit (P0011, Beyotime, Shanghai, China). Lysate supernatant (20 μg) was added to the 10% SDS-PAGE gel for electrophoresis (Constant Voltage = 120 V), and the proteins in the gel were transferred to a PVDF membrane (Constant Current = 400 mA, wet transfer, 1 h for high molecular weight proteins and 40 min for small molecular proteins). Blocking was performed using 5% skim milk, followed by the addition of primary antibodies and incubation at 4 °C overnight with the following primary antibodies: Smad3 (1:1000, PA5-32588, Invitrogen, Carlsbad, CA, USA), p-Smad3 (1:1000, 9520S, CST, Danvers, MA, USA), GAPDH (1:5000, 60004-1-Ig, Proteintech, Wuhan, China), FLAG (1:1000, F3165-1MG, SIGMA, St. Louis, MO, USA), Snail (1:1000, 3879S, CST, Danvers, MA, USA), Fn (1:1000, ab2413, Abcam, Cambridge, MA, USA), P-smad3 (1:1000, SC-517575, Santa Cruz, CA, USA), LAMIN A/C (1:1000, 4777S, CST, Danvers, MA, USA), NP (1:1000, GTX125989, Genetex, San Antonio, USA). After adequate incubation, the primary antibody was removed and washed with TBST, followed by adding 3% skim milk containing horseradish peroxidase (HRP)-conjugated secondary antibody (1:1000, Jackson ImmunoResearch Laboratories, West Grove, PA, USA) for 1 h at room temperature. After adequate washing, the membranes were incubated in ECL reagent (Bio-Rad, Hercules, CA, USA) and exposed using a chemical illuminance imaging system (Tanon, Shanghai, China).

### 4.6. Quantitative Real-Time PCR (qPCR)

Total RNA in cells and tissues was extracted using the Ultrapure RNA kit (Co Win Biotech, Beijing, China). Total RNA was reverse-transcribed to cDNA using HiScript III RT SuperMix. The qPCR reaction system consisted of 5 μL iTaq^TM^ Universal SYBR Green Supermix (Bio-Rad), 2.5 μL H_2_O, 0.25 μL of each forward and reverse primer (10 μM), and 1 μL of a sample. Using the CFX Connect^TM^ platform (Bio-Rad), the amplification program was 1 cycle at 95 °C for 3 min, 39 cycles at 95 °C for 10 s, 60 °C for 10 s and 72 °C for 20 s, and 1 cycle at 95 °C for 10 s. The primers used are shown in Table S1. Gene expression quantification was performed using the 2^−ΔCT^ method, with GAPDH serving as the housekeeping gene.

### 4.7. Cell Counting Kit-8 (CCK8) Assay

Cells were seeded in 96-well plates at a density of 3 × 10^4^ cells, cultivated for 24 h in an incubator at 37 °C and 5% CO_2_. On the following day, cells were treated with various concentrations of HBT. After 24 h, the medium was removed, and the cells were washed twice with PBS. After discarding the excess liquid, a fresh medium containing 10% CCK8 reagent was added. Then, 96-well plates were incubated in a 37 °C incubator in the dark for a certain period and then detected by a multifunctional microplate reader.

### 4.8. Nuclear and Cytoplasmic Extraction

The procedures for cell infection and drug treatment remain consistent with previous protocols. After collecting the cells, a nuclear–cytoplasmic extraction kit (78835, Thermo Fisher Scientific, Waltham, MA, USA) was utilized for protein extraction. Finally, a loading buffer was added in preparation for subsequent Western blot analysis.

### 4.9. Immunohistochemistry

Lung tissues embedded in paraffin were sectioned at 3 µm thickness. Sections were deparaffinized using xylene and hydrated with various concentrations of alcohol (100%, 95%, 90%, 80%, and 70%) in descending order. Endogenous peroxidase activity was inactivated by a 3% H_2_O_2_ solution. Sections were subjected to antigen retrieval by heating in 10 mM sodium citrate solution (pH = 6.0). Subsequently blocked by a 5% BSA solution. Then, sections were incubated overnight at 4 °C with the primary antibody diluted in 1% BSA for 24 h. Dilution ratios were established of primary antibody TGF-β1 (1:100, bs-0086R, Bioss, Woburn, MA, USA), Fn (1:100, ab2413, Abcam, Cambridge, MA, USA), and collagen I (1:100, 1310-01, SouthernBiotech, Birmingham, AL, USA). Sections were incubated with HRP-labeled goat anti-mouse/rabbit IgG polymer (DAKO, K4001/K4003) or secondary antibodies diluted in 1% BSA for 1 h at room temperature. Color development was performed using 3,3′-diamino-benzidine (DAB) substrate on the sections. After nuclear staining with hematoxylin, the sections were mounted with neutral balsam. Sections were scanned with the use of a slide Scan Analysis Imaging system (C9600-12, Hamamatsu, Japan) for further analysis.

### 4.10. Plaque Assay

A plaque assay was performed to measure virus titers. MDCK cells were seeded into 12-well plates at a density of 3 × 10^5^ cells for viral plaque detection. On the second day post-inoculation, cells were washed twice with PBS. Diluted cell supernatants were then added to each well (The A549 supernatant was diluted 1 × 10^2^ fold. The MDCK supernatant was diluted 1 × 10^4^ fold.), shaken briefly, and incubated at 37 °C. After 2 h, the medium in each well was discarded. The 0% FBS DMEM containing 1% low-melting point agarose and 2.5 μg/mL TPCK-treated trypsin was prepared and overlaid onto the cells. The plates were incubated at 37 °C for 3–4 days. After incubation, the agarose was fixed by adding 4% paraformaldehyde to the wells. The agarose gels were then removed, and the plaques were stained with 1% crystal violet.

### 4.11. Molecular Docking

The 3D structure of herbacetin was downloaded in SDF format from the PubChem database and converted to PDB format using OpenBabel v3.1.1 [46]. The prepare_ligand command from AutoDock Vina v1.1.2 was used to add hydrogens and assign charges to the compound, generating a PDBQT file [47]. The rotatable single bonds of herbacetin were automatically identified and labeled. The PDB file of N1 neuraminidase, obtained from the UniProt database, was used as the receptor. Water molecules and sulfate ions were removed from the protein structure using PyMOL v3.0. After hydrogenation and charge assignment using the prepare_receptor4 command, the receptor file was saved in PDBQT format. The DoGSiteScorer algorithm in Proteins Plus (https://proteins.plus/, 8 October 2024) was employed to predict protein pocket sites, and the pocket sizes and coordinates are shown in Table S2 [48]. Finally, each protein pocket parameter and the three AutoDock Vina run parameters (energy_range = 5, exhaustiveness = 8, and num_modes = 8) were saved in a “config.txt” file. In command-line mode, the Lamarckian genetic algorithm was applied to run AutoDock Vina, docking the compound ligands with the protein pockets. The docking scores were recorded and analyzed.

### 4.12. Biolayer Interferometry (BLI) Assay

The interaction between HBT and NA (N1, PR8) proteins was examined using a BLI assay. Super streptavidin biosensors (SSA) were purchased from Sartorius (Gottingen, Germany), NA protein was purchased from Sino Biological (Beijing, China), and a biotinylated labeling kit (Genemor, G-MM-IGT) was used for protein labeling. Finally, the protein was resuspended using PBS, and 0.02% Tween 20/1% DMSO/PBS buffer was used to dissolve proteins (20 μg/mL) and dilute HBT. The NA protein was immobilized onto the sensor surface using the instrument, and ligand binding interactions were subsequently monitored in real-time.

### 4.13. Cellular Thermal Shift Assay (CETSA)

Cells were seeded in 10 cm dishes at a density of 6 × 10^6^ cells and transfected with FLAG-tagged NA plasmids. The drug or DMSO was added four hours before sample collection. After sampling, the cells were rinsed once with PBS, collected by centrifugation, and resuspended in PBS with phosphatase inhibitors and protease inhibitors. A volume of 100 µL of cell suspension was transferred into EP tubes and subjected to heating at temperatures ranging from 41 °C to 63 °C for 3 min using a PCR instrument. Subsequently, the cells were lysed through three freeze–thaw cycles in liquid nitrogen, followed by a water bath at 37 °C. The lysate was then centrifuged at 20,000× *g* for 20 min at 4 °C to obtain the protein supernatant for subsequent Western blot analysis.

### 4.14. Neuraminidase Inhibition Assay

The NA inhibitor experiments were conducted using the NA-Fluor^TM^ Influenza Neuraminidase Assay Kit (4457091, Thermo Fisher Scientific, Waltham, MA, USA). Using the instructions provided, a 1% Triton X-100 solution was utilized to inactivate the virus for detection purposes. A standard curve was generated using varying concentrations of 4-methylumbelliferone sodium salt. The appropriate volume of viral lysate for experimentation was determined by diluting the lysate at different ratios.

For the inhibitor assays, HBT was diluted to various concentrations using DMSO. The drug and viral lysate were pre-mixed and incubated at 37 °C for 30 min. Subsequently, a fluorescent substrate was added and allowed to incubate for 1 h before adding a stop solution for detection. OSE (10 μM) was used as a positive control.

### 4.15. HE and Masson Staining

For HE staining, the tissue sections were deparaffinized in xylene and rehydrated through a graded alcohol series. The sections were stained with hematoxylin for 3–5 min, followed by differentiation in a differentiation solution, and rinsed with water. Eosin staining was then applied for 5 min. Following staining, the sections were dehydrated in 85% and 95% alcohol for 5 min and then mounted with neutral resin after being cleared in xylene.

For Masson’s trichrome staining, the sections were deparaffinized in xylene and rehydrated through graded alcohol concentrations. The sections were incubated overnight in Masson staining solution 1, followed by washing with water until the sections were colorless. The sections were then stained with Masson solution 2 for 3–5 min and rinsed 2–3 times with water. Next, the sections were placed in Masson solution 3 for 30 s to 1 min, then stained with Masson solution 4 for 5–20 s. The sections were differentiated in 1% glacial acetic acid for a few seconds, followed by dehydration in absolute ethanol and n-butanol, and finally mounted with neutral resin.

### 4.16. Statistical Analysis

Statistical analysis of the data was performed using GraphPad Prism 8 software. All data are shown as the mean ± SD. The *t*-test was used for statistical analysis of two groups of data, and one-way ANOVA with Turkey’s test was used for the statistical analysis of more than two groups of one-factor data. *p*-values of less than 0.05 were considered to indicate a statistically significant difference.

## 5. Conclusions

In summary, this study demonstrates that lung injury induced by the influenza virus can progress to PF and that NA proteins play a crucial role in its development. HBT inhibits influenza virus infection by targeting NA proteins and exerts a pharmacological effect in treating lung injury and PF caused by IAV. Targeting NA proteins is emphasized as a critical strategy for the future development of inhibitors aimed at treating influenza-induced PF.

## Data Availability

The original contributions presented in this study are included in the article/Supplementary Materials. Further inquiries can be directed at the corresponding authors.

## Supplementary Materials

The following supporting information can be downloaded at https://www.mdpi.com/article/10.3390/ph18091306/s1: Figure S1. Gene expression analysis of influenza-infected mouse lung tissue was obtained from the GEO database. Figure S2. CCK-8 results of HBT on A549 and MDCK cells. Figure S3. Results of the NA enzyme activity of H3N2, H9N2, and IBV inhibited by HBT. Table S1. List of primer sequences used. Table S2. 50 binding pockets of neuraminidase (N1) used.

## Abbreviations

The following abbreviations are used in this manuscript:

IAV	Influenza A virus
IBV	Influenza B virus
ARDS	acute respiratory distress syndrome
PF	pulmonary fibrosis
*R. rosea* L.	*Rhodiola rosea* L.
HBT	Herbacetin
BLI	BioLayer Interferometry
CETSA	cell thermal shift assay
NA	neuraminidase
TGF-β	transforming growth factor-β
Fn	fibronectin
TNF-α	tumor necrosis factor-α
OSE	oseltamivir
DMSO	Dimethyl sulfoxide
AIV	Avian Influenza virus
RBV	ribavirin
MOI	Multiplicity of infection
MDCK	Madin-Darby Canine Kidney
PBS	phosphate-buffered saline
qPCR	Quantitative Real-Time PCR
CCK8	Cell Counting Kit-8
DAB	diamino-benzidine
