# Supplementary material for: Herbacetin Alleviates Influenza Virus-Induced Lung Injury and Fibrosis by Targeting the Neuraminidase Protein
Source: Pharmaceuticals (Basel). 2025 Aug 30;18(9):1306. doi: 10.3390/ph18091306 (PMC12473129; doi:10.3390/ph18091306)

Supplement material

Method and matterial

1. Gene expression analysis

The keywords "influence" and "lung tissue" were submitted to the database of GEO (http://www.ncbi.nlm.nih.gov/geo/). The screening criteria for Gene Expression Omnibus Series (GSE) are as follows: (1) the series includes both the infected group and the healthy control group; (2) a minimum of 10 samples must be included; (3) lung samples are required for sequencing. Ultimately, a set of samples derived from mice (GEO number = GSE119853, Influenza = 8, Control = 10) was selected for transcriptomic analysis. For data processing, R (version 4.3.1) was employed for data standardization, correction, and gene name annotation. Principal Component Analysis (PCA), crucial for evaluating the effectiveness of transcriptomic data, enables researchers to identify patterns, outliers, and potential batch effects and discern whether biological replicates cluster together. Finally, the expression levels of key genes were verified and presented as box plots.

2. List of primer of qPCR

Table S1. List of primer sequences used

Primer	Sequence
Hu-FN-F	TCAGCTTCCTGGCACTTCTG
Hu-FN-R	TCCCTGGGGATGTGACCAAT
Hu-SNAIL-F	TCGGAAGCCTAACTACAGCGA
Hu-SNAIL-R	AGATGAGCATTGGCAGCGAG
Hu-GAPDH-F	AAGGCTGTGGGCAAGG
Hu-GAPDH-R	TGGAGGAGTGGGTGTCG
Mou-Fn-F	AGACCATACCTGCCGAATGTAG
Mou-Fn-R	GAGAGCTTCCTGTCCTGTAGAG
Mou-snail-F	GTCCAGCTGTAACCATGCCT
Mou-snail-R	TGTCACCAGGACAAATGGGG
Mou-GAPDH-F	GGTGAAGGTCGGTGTGAACG
Mou-GAPDH-R	CTCGCTCCTGGAAGATGGTG
IAV-NP-F	CATCTTTCTGGCACGGTCTG
IAV-NP-R	GGCTACTGCAGGTCCATACA

3. The binding pocket of neuraminidase (N1) used in this study.

Table S2. 50 binding pockets of neuraminidase (N1) used

Pocket Center pocket	X	Y	Z	Max Radius
Pocket_Center_0	-24.66	51.66	109.53	18.49
Pocket_Center_10	31.45	49.42	106.26	10.36
Pocket_Center_11	21.73	46.81	115.42	13.1
Pocket_Center_12	10.32	21.65	116.07	10.11
Pocket_Center_13	29.27	59.37	116.75	10.14
Pocket_Center_14	-27.48	40.22	115.45	9.75
Pocket_Center_15	17.02	72.66	101.71	10.7

Pocket_Center_16	16.82	15.47	103.95	11.87
Pocket_Center_17	-33.3	34.7	102.44	10.83
Pocket_Center_18	-8.41	77.89	116.12	8.8
Pocket_Center_19	-13.54	83.66	103.95	11.55
Pocket_Center_1	2.32	76.41	109.28	18.96
Pocket_Center_20	19.59	73.58	87.77	9.28
Pocket_Center_21	-7.92	56.11	105.8	10.6
Pocket_Center_22	-4.88	40.51	105.7	10.86
Pocket_Center_23	-5.17	71.43	87.98	9.95
Pocket_Center_24	8.7	28.43	88.3	9.24
Pocket_Center_25	23.05	57.03	88.53	8.74
Pocket_Center_26	24.81	34.36	101.04	11.36
Pocket_Center_27	-6.62	64.73	104.7	12.18
Pocket_Center_28	0.79	50.19	115.35	7.37
Pocket_Center_29	-19.99	43.47	87.73	9.01
Pocket_Center_2	0.07	23.66	108.95	18.9
Pocket_Center_30	-21.11	53.33	81.94	6.9
Pocket_Center_31	15.7	57.88	105.09	11.89
Pocket_Center_32	7.78	61.07	105.4	7.69
Pocket_Center_33	-14.41	26.19	100.31	11.2
Pocket_Center_34	-1.46	27.14	82.68	7.3
Pocket_Center_35	10.05	35.31	103.02	8.58
Pocket_Center_36	-13.92	40.97	102.41	7.7
Pocket_Center_37	24.56	46.8	83.18	6.89
Pocket_Center_38	12.18	43.65	105.43	7.6
Pocket_Center_39	11.67	11.98	84.67	7.95
Pocket_Center_3	9.63	62.47	93.52	10.11
Pocket_Center_40	-23.21	67.84	101.23	9.85
Pocket_Center_41	11.76	67.09	83.66	5.67
Pocket_Center_42	-45.2	52.64	96.11	6.09
Pocket_Center_43	-1.18	3.66	96.54	5.96
Pocket_Center_44	4.69	73.15	84.99	6.73
Pocket_Center_45	-33.37	36.84	85.38	6.52
Pocket_Center_46	47.71	47.4	98.02	5.95
Pocket_Center_47	-22.25	20.9	97.96	6.23
Pocket_Center_48	4.08	96.36	97	6.02
Pocket_Center_49	-27.83	73.62	98.6	5.91
Pocket_Center_4	-8.32	36.83	92.65	10.04
Pocket_Center_5	-20.15	65.04	85.3	12.2
Pocket_Center_6	-10.96	58.73	93.42	10.64
Pocket_Center_7	-13.42	28.58	85.16	12.05
Pocket_Center_8	13.91	41.64	93.71	10.78
Pocket_Center_9	23.25	35.29	85.94	11.87

Result

Figure S1.TGF-β pathway related genes are increased in lung tissues of influenza infection. A. The gene expression profiles of lung tissues of mice infected with influenza in the GEO database were analyzed. PCA plot illustrating the distinction between the influenza infection group (blue area) and the control group (red area). B. Gene expression analysis graph displaying the differences in expression levels of col1a1, col3a1, fn, snail, tgfb1, tgfbr1, and

tgfbr2 between the influenza infection group and the blank control group, with *p*-values indicated below their respective gene names.

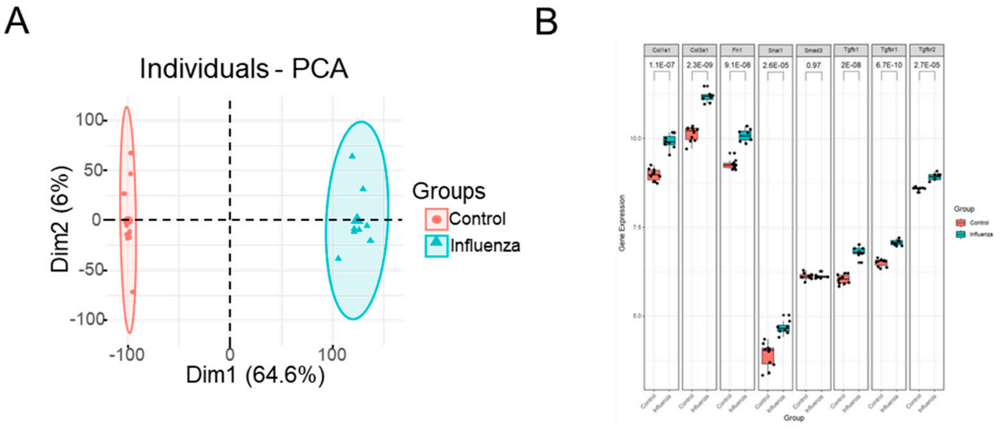

Figure S2. The effect of HBT on cell viability. Different concentrations of HBT were added to the cells and cell viability was then measured using CCK8.

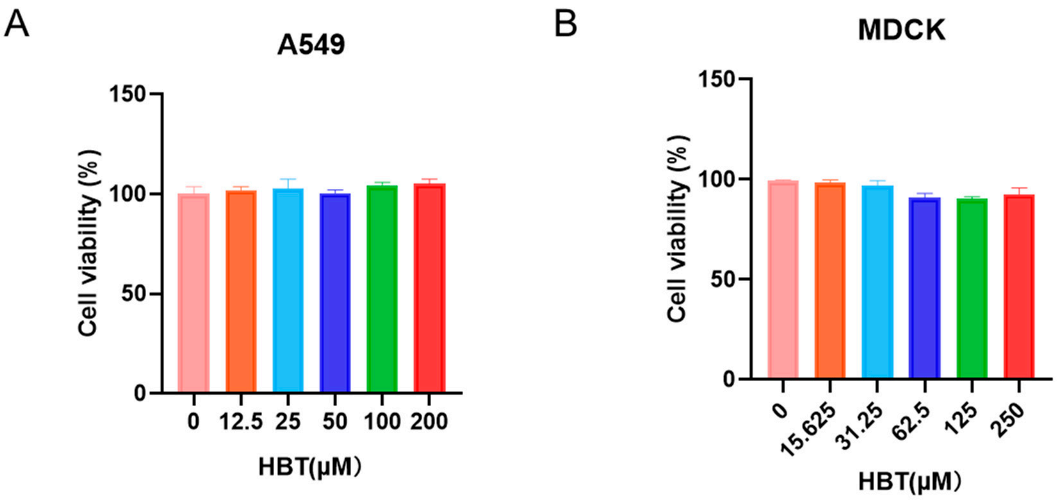

Figure S3. Results of NA enzyme activity of H3N2, H9N2, IBV inhibited by HBT. HBT was diluted with DMSO to prepare different concentrations for detection. OSE at a concentration of 10 μM was used as a positive control.

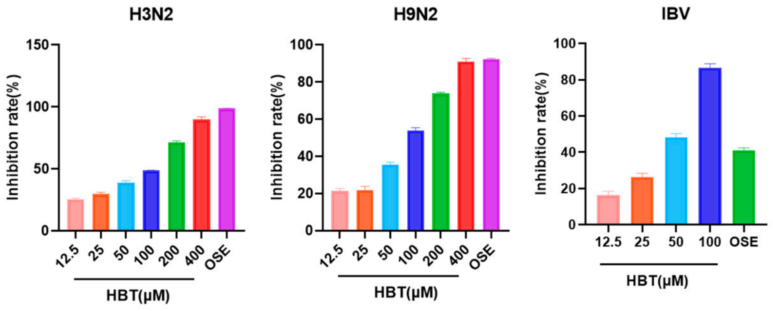

Supplement: Supplementary file 1 [file pharmaceuticals-18-01306-s001.zip › pharmaceuticals-3791123-supplementary.pdf]
